# Complete Genome Sequences of 16 Canadian Strains of *Salmonella enterica* subsp. *enterica* Serovar Enteritidis

**DOI:** 10.1128/genomeA.00330-14

**Published:** 2014-04-24

**Authors:** Muhammad A. Rehman, Kim Ziebell, John H. E. Nash, Andrew M. Kropinski, Ashley Ross, Mariam Al-Lami, Patrick Boerlin, Linda Chui, John Devenish, Sadjia Bekal, Morag Graham, Kingsley K. Amoako, Roger P. Johnson

**Affiliations:** a Laboratory for Foodborne Zoonoses, Public Health Agency of Canada, Guelph, Ontario, Canada; b Department of Pathobiology, Ontario Veterinary College, University of Guelph, Guelph, Ontario, Canada; c Alberta Provincial Laboratory for Public Health, Edmonton, Alberta, Canada; d Department of Laboratory Medicine and Pathology, University of Alberta, Edmonton, Alberta, Canada; e Animal Health Microbiology Laboratory, Canadian Food Inspection Agency, Ottawa, Ontario, Canada; f Laboratoire de Santé Publique du Québec, Sainte-Anne-de-Bellevue, Quebec, Canada; g National Microbiology Laboratory, Public Health Agency of Canada, Winnipeg, Manitoba, Canada; h Department of Medical Microbiology, University of Manitoba, Winnipeg, Manitoba, Canada; i Canadian Food Inspection Agency, National Centers for Animal Disease, Lethbridge, Alberta, Canada

## Abstract

*Salmonella enterica* subsp. *enterica* serovar Enteritidis is an important zoonotic food-borne pathogen causing serious human illnesses frequently linked to poultry products. Here, we report fully assembled genome sequences of 16 *S*. Enteritidis strains with common pulsed-field gel electrophoresis (PFGE) and phage types (8, 13, 13a, and 14b) that predominate in North America.

## GENOME ANNOUNCEMENT

Nontyphoidal *Salmonella* infections represent a major food-borne threat, causing an estimated 93.8 million cases and 155,000 deaths globally each year (1). *Salmonella enterica* subsp. *enterica* serovar Enteritidis ranks among the most common serovars associated with food-borne illness in many countries, with contaminated eggs and poultry considered the most frequent sources. Over the past decade, the overall incidence of salmonellosis in Canada has remained relatively constant, but between 2003 and 2008, the reported *S*. Enteritidis infections increased almost 3-fold, from 2.2 to 6.2 per 100,000 persons per year (2, 3), and in 2011, they accounted for 40.6% of all reported cases of human salmonellosis (4). The predominant phage types (PTs) during this period were 8, 13, and 13a (2, 3). Because *S*. Enteritidis is genetically highly homogenous, subtyping by current pulsed-field gel electrophoresis (PFGE) and phage typing methods is of limited value. Whole-genome sequencing (WGS) might overcome these limitations in subtyping by providing the discriminatory power needed to differentiate highly clonal *S*. Enteritidis strains (5). However, only one finished *S*. Enteritidis genome sequence (PT4, strain P125109) (6) is currently available in public databases for comparison.

Here, we report the fully closed genome sequences of 16 strains of *S*. Enteritidis isolated from eight clinical, two chicken farm environmental, one animal, and five food sources at diverse locations within Canada, which belong to PTs 8 (*n* = 4), 13 (*n* = 5), 13a (*n* = 6), and 14b (*n* = 1).

Genomic DNA was extracted using the Qiagen EZ1 DNA tissue kit (catalog no. 953034). Sequencing was performed on two platforms: (i) Roche 454 GS-FLX Titanium (at McGill University and Génome Québec, Québec, Canada), achieving >40× average genome coverage, and (ii) Illumina HiSeq 2500 (Centre for Applied Genomics, Hospital for Sick Children, Toronto, Ontario, Canada) using the TruSeq DNA sample preparation kit (Illumina), with 2 × 101 paired-end runs achieving >90× average genome coverage. The reads were analyzed and quality checked using FastQC (http://www.bioinformatics.babraham.ac.uk/projects/fastqc/). Genome assemblies were created by using the MIRA assembler version 4.0 (7) and by manually checking potential joins using the Gap5 software of the Staden package (8). Aligning the contigs to the closely related genome of *S*. Enteritidis strain P125109 (GenBank accession no. AM933172) and, for some strains, comparison with their optical maps (9), together with the finishing process, produced fully assembly genomes. These consisted of single-chromosome contigs ranging from ~4,684,342 to 4,753,867 bp, with an average G+C content of ~52.17%. The genomes were annotated with the National Center for Biotechnology Information (NCBI) Prokaryotic Genomes Annotation Pipeline (PGAP) (http://ncbi.nlm.nih.gov/genomes/static/Pipeline.html), identifying an average of ~4,500 coding DNA sequences (CDS) per genome. No attempt was made to identify the plasmid sequences in the genomes.

These 16 closed *S*. Enteritidis genome sequences have been deposited in GenBank. Their availability will provide opportunities for high-resolution investigation of properties of *S*. Enteritidis that are informative about transmission, virulence, evolution, and discovery of markers for reliable subtyping that are required for outbreak detection and source attribution. Further information and analyses of these isolates will be included in a forthcoming publication.

### Nucleotide sequence accession numbers.

The complete genome sequences of these *S*. Enteritidis strains are available in GenBank under BioProject no. 219482 and the GenBank accession numbers listed in Table 1.

**TABLE 1:** Accession and isolate numbers for the 16 *Salmonella* Enteritidis strains sequenced in this study

GenBank accession no.	Isolate accession no.	Original isolate no.
CP007175	EC20110354	M10MD013186
CP007245	EC20120008	SE20060017
CP007246	EC20100101	SA02DT09081501
CP007247	EC20110221	NML 5-6746
CP007248	EC20090698	SA20090217
CP007250	EC20110355	M09MD3451
CP007251	EC20110353	M10MD12420
CP007252	EC20111175	11 SU008 7-10
CP007253	EC20111174	11 SU 006 4-8
CP007254	EC20111095	110672
CP007258	EC20110360	M04MD5221
CP007259	EC20110359	M04MD2595
CP007260	EC20110358	M09MD011202
CP007261	EC20110357	M03ER103
CP007262	EC20110356	M09MD3812
CP007263	EC20110361	M09MD11210
